# The use of targeted exome sequencing in genetic diagnosis of young patients with severe hypercholesterolemia

**DOI:** 10.1038/srep36823

**Published:** 2016-11-10

**Authors:** Long Jiang, Wen-Feng Wu, Li-Yuan Sun, Pan-Pan Chen, Wei Wang, Asier Benito-Vicente, Fan Zhang, Xiao-Dong Pan, Wei Cui, Shi-Wei Yang, Yu-Jie Zhou, Cesar Martin, Lu-Ya Wang

**Affiliations:** 1 Department of Atherosclerosis, Beijing An Zhen Hospital, Capital Medical University, Beijing Institute of Heart, Lung and Blood Vessel Diseases, 2 Anzhen Road, Chaoyang District, Beijing, 100029, China; 2 Department of Cardiovascular, the Second Affiliated Hospital of Nanchang University, Nanchang, 330006, Jiangxi Province, China; 3 Department of Emergency Intensive Care Unit, Beijing AnZhen Hospital, Capital Medical University, Beijing, 100029, China; 4 Department of Dermatology, Beijing AnZhen Hospital, Capital Medical University, Beijing, 100029, China; 5 University of South China, Hengyang, 421001, China; 6 The Affiliated Hospital of North China University of Science and Technology, North China University of Science and Technology, Tangshan, 063000, Hebei Province, China; 7 Biofisika Institute (UPV/EHU, CSIC) and Dpt. Biochemistry and Molecular Biology, University of the Basque Country, UPV/EHU, Spain; 8 School of Computer Engineering, Nanyang Technological University, 639798, Singapore

## Abstract

Familial hypercholesterolemia (FH) is an autosomal dominant disorder. Although genetic testing is an important tool for detecting FH-causing mutations in patients, diagnostic methods for young patients with severe hypercholesterolemia are understudied. This study compares the target exome sequencing (TES) technique with the DNA resequencing array technique on young patients with severe hypercholesterolemia. A total of 20 unrelated patients (mean age 14.8 years) with total cholesterol > 10 mmol/L were included. 12 patient samples were processed by DNA resequencing array, 14 patient samples were processed by TES, and 6 patient samples were processed by both methods. Functional characterization of novel mutations was performed by flow cytometry. The mutation detection rate (MDR) of DNA resequencing array was 75%, while the MDR of TES was 100%. A total of 27 different mutations in the LDLR were identified, including 3 novel mutations and 8 mutations with previously unknown pathogenicity. Functional characterization of c.673delA, c.1363delC, p.Leu575Phe and p.Leu582Phe variants found that all of them are pathogenic. Additionally, 7 patients were diagnosed with Heterozygous FH (HeFH) in which lipid levels were significantly higher than common HeFH patients. This data indicates that TES is a very efficient tool for genetic diagnosis in young patients with severe hypercholesterolemia.

Familial hypercholesterolemia (FH, OMIM: #143890) is a common autosomal dominant disorder that causes severe elevations in total cholesterol (TC) and low-density lipoprotein cholesterol (LDL-C)^1^. FH is primarily associated with premature coronary heart disease, raised serum cholesterol levels, and xanthoma. The most common cause of FH is a mutation in one of the following three genes: *low-density lipoprotein receptor* (*LDLR*, MIM #606945), which accounts for nearly 90% of causal mutations; *apolipoprotein B* (*APOB*; MIM# 107730), and *proprotein convertase subtilisin/kexin type 9* (*PCSK9*; MIM# 607786)^2^. Current evidence indicates that FH may cause a serious public genetic burden because of the high prevalence of heterozygous FH (HeFH) compared to homozygous FH (HoFH). While the global prevalence of HeFH is between 1 in 200 to 1 in 500 individuals, the prevalence of HoFH is between approximately 1 in 160,000 to 1 in 300,000 individuals^3^. Although early LDL-c lowering therapies, such as statins, can improve FH survival, FH remains underdiagnosed and undertreated in most countries^4^. Recently, a consensus statement of FH experts emphasized the need for a global effort to generate and integrate data to better manage FH because of the challenge it poses to global public health^5^.

Conventional genetic diagnosis of FH is primarily based on direct DNA sequencing or a combination of direct sequencing with use of the multiplex ligation-dependent probe amplification (MLPA), detects large insertion or deletion mutations^6^. Because of the high cost of both these methods, alternative DNA resequencing assays have been developed to optimize the detection of FH-causing mutations^7^. In recent years, next-generation sequencing has also been successfully used to conduct screens for FH-causing mutations^8^,^9^. We previously showed that target exome sequencing (TES) successfully detected LDLR mutations in a FH patient^10^. The aim of the present study is to compare the TES technique with the DNA resequencing array technique in the genetic diagnosis of young patients with severe hypercholesterolemia. We found that TES-based screenings yields a more accurate genetic diagnosis and we conclude that TES is the more appropriate diagnostic technique for young patients with severe hypercholesterolemia.

## Results

### Study subjects

A total of 20 probands were included in our study. The mean age of the probands was 14.8 ± 8.8 years. 10 males and 10 females were included in our study. About 60% probands were from South China. All the probands presented xanthoma and 85% had corneal arcus. Clinical and biochemical characteristics of the probands are shown in Table 1. The mean TC and LDL-C levels were 16.1 ± 2.9 mmol/L and 13.2 ± 2.4 mmol/L, respectively. Color Doppler ultrasound showed that half of the probands had regurgitation of mitral valve and 55% of the probands had regurgitation of aortic valve. The mean IMT of ten probands was 0.18 cm and the mean CVFR of 12 probands was 2.19. Nearly 90% of the probands had statin treatment and only one proband had no lipid lowering treatment because of parental refusal.

### Mutation detection rates

**Figure 1:**
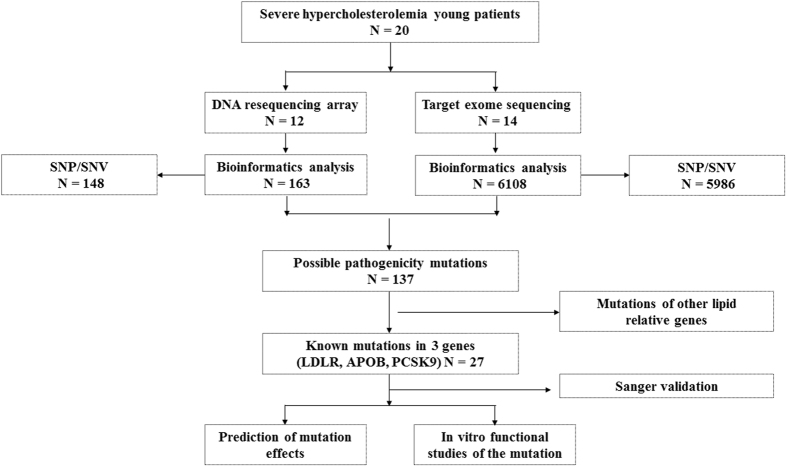
The flow chart of mutations screening.

20 proband DNA samples were analyzed by DNA resequencing array and target exon sequencing (Fig. 1). A total of 27 mutations in LDLR gene were identified (Table S1). There was no mutation found by DNA resequencing array in proband 11, even though a c.1187–10 G>A mutation was detected by the whole-exome sequencing method in a previous study^11^. In the DNA array group, a total of 16 mutations were identified across the 12 probands tested with DNA array. However, the DNA resequencing array method only detected 12 mutations in these probands, yielding a detection rate of 75%. In TES group, all 23 mutations were detected across 14 probands, with a detection rate of 100%. For the 6 proband DNA samples studied by both methods, DNA resequencing array correctly detected 72.7% (8/11) of mutations, while TES correctly detected 100% (11/11) of mutations (Table 2).

### Spectrum of FH-related mutations

Overall, 27 different LDLR mutations were found across the 20 probands (Table 2). In Proband 12, only one SNP (1060 + 10 G > C in intron 7, rs12710260) was detected. No mutations in apoB and PCSK9 genes were found in any of the probands. From the 27 mutations detected, there were 19 missense mutations (70.4%), 2 nonsense mutations (7.4%), 2 single base deletion mutations (7.4%), 3 splicing-site mutations (11.1%), and 1 synonymous mutation (3.7%). Most mutations were located in either exon 4 (7/27, 25.9%) or exon 12 (5/27, 18.5%). The most common mutation was W483X (n = 6, 22.2%), which is located in the tenth exon of the LDLR gene, in which a G-to-A substitution at nucleotide 1448 introduces a premature termination codon leading to a truncated LDLR. The second most common mutation detected was A627T (n = 2, 7.4%), which is located in the thirteenth exon encoding the EGF precursor homology domain of LDLR. It has been previously shown that LDLR^A627T^ is a pathogenic variant that accumulates inside the cell and is unable to enter the plasma membrane^12^.

### Novel mutations and mutations with unknown pathogenicity found in the LDLR gene

We found a total of 3 novel mutations in *LDLR* and 8 previously described mutations with unknown pathogenicity (Table S2). The 3 novel mutations were: c.673delA, p.Leu575Phe, and p.Leu582Phe. The mutation c.673delA causes a frame shift, leading to a premature stop codon 40 amino acids after the deletion site. *In silico* analysis predicted this mutation to be pathogenic (Table S2). *In silico* analysis of p.Leu575Phe, and p.Leu582Phe variants showed some discrepancies depending on the software used. While align GVGD predicts p.Leu575Phe and p.Leu582Phe to be probably damaging, the other *in silico* software predicts both as pathogenic.

The 8 previously reported mutations with unknown pathogenicity found in this study were: p.Asp172His, p.Cys204Tyr, p.Cys222Phe, c.818–2 A > G, p.Arg416Trp, c.1363delC, p.Cys711Tyr, and p.Asn825Lys. *In silico* analysis predicted all of these mutations to be pathogenic. Proband carrying the p.Asn825Lys variant showed the highest CADD value of 21.3; the lowest CADD value (7.147) was found in the proband carrying p.Cys222Phe.

### Functional studies

**Figure 2:**
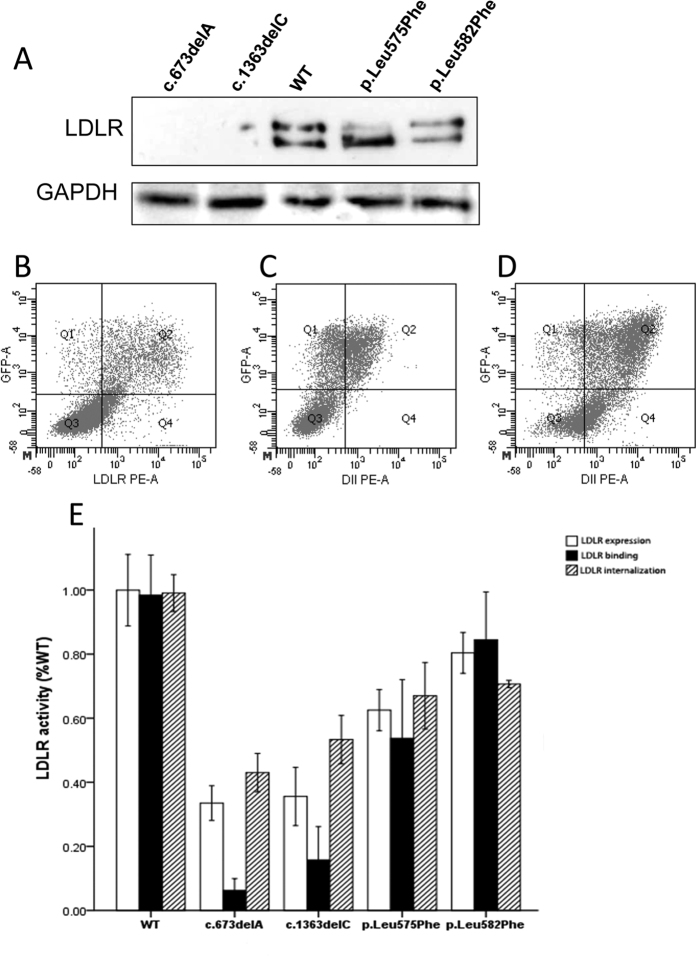
Functional characterization of novel LDLR variants. (**A**). LDLR expression. Cells were transfected with the corresponding plasmids carrying the mutations of interest, LDLR was overexpressed for 48 h and then cells were analyzed by Western blot. Whole cell extracts (20 μg) were fractioned in non reducing 8.5% SDS-PAGE, transferred onto nitrocellulose membranes for incubation with a rabbit polyclonal anti-hLDLR antibody and detected by chemioluminiscence as described in Methods section. A representative experiment from three independently performed assays is shown in upper panel. (**B–D**): Analysis of variant functionality by FACS: LDLR expression at cellular membrane (using WT as example, B); LDL binding after 4 h incubation at 4 °C (using WT as example, C); and LDL internalization efficiency after 4 h incubation at 37 °C (using WT as example, D). (**E**) Functional characterization of LDLR variants. LDLR expression, LDL binding and LDL uptake. 10,000 cells were acquired in a Facscalibur and values of LDL uptake, binding and LDLR expression were calculated as described in Methods. The values represent the mean of triplicate determinations (n = 3); error bars represent ± SD.

We experimentally characterized functionality in the 3 novel LDLR variants identified in this study (c.673delA, p.Leu575Phe, and p.Leu582Phe) and in c.1363delC, a novel variant previously found by our group^13^. A total of five plasmids carrying EGFP were constructed (WT, c.673delA, c.1363delC, p.Leu575Phe and p.Leu582Phe) as described in Materials and Methods. LDLR expression was assayed by immunoblotting and, as shown in Fig. 2A (upper panel), c.673delA, c.1363delC variants are not expressed in transfected cells while expression of mature p.Leu575Phe and p.Leu582Phe variants is lower than WT LDLR. Equal loading of protein was confirmed in each blot by membrane stripping and further incubation with antibodies to visualize cytosolic GAPDH protein (Fig. 2A, lower panel).

Analysis by cytometry was performed to determine activity of these variants, examples of FACS raw results are shown in Fig. 2B–D. We found that c.673delA and c.1363delC resulted in diminished expression (approximately 65% of WT). To a lesser extent, LDLR expression in p.Leu575Phe, and p.Leu582Phe variants was also diminished (Fig. 2E). Concurrently, LDL binding and uptake were both also significantly diminished in all 4 variants, and more markedly in c.673delA and c.1363delC variants (Fig. 2E).

### Relationship between genotype and phenotype

A cascade screening in 18 families including a total of 71 patients was performed and the obtained data is shown in Table 3. Among them, genetic diagnosis indicates that there are 4 HoFH patients, 14 compound heterozygous patients, 52 HeFH patients (included 7 HeFH with high TC) and 1 negative mutation patient. The average age of HoFH and compound heterozygous patients was lower than the average age of HeFH patients. However, the values of the lipid profile in HeFH patients (TC, LDL-C, and non-HDL-c) were lower than those determined in HoFH and compound heterozygous patients (Table 3). Lipid profile comparision between HoFH and compound heterozygous patients showed no significant differences between the two groups. Interestingly, 7 of the patients diagnosed with HeFH had significantly higher lipid levels than the other HeFH patients. These seven patients had higher TC, LDL-C and non-HDL-C levels than the remaining patients in the HeFH group. Based on the different genotypes of HeFH found in this study, a comparison analysis of LDL-C was assayed between three different groups: patients with missense mutations, patients with nonsense mutations, and patients with splicing-site mutations. Lipid analysis found that patients with nonsense mutations had significant lower LDL-C level than patients with missense mutations. There were no significant differences between the levels of the other lipids that were analyzed (Table S3).

## Discussion

In this study, we determined that target exome sequencing is a powerful tool for genetic diagnosis of young patients with severe hypercholesterolemia. We used both DNA resequencing array and target exon sequencing and found 27 LDLR mutations. Among them, 3 were novel mutations and 8 were previously described LDLR mutations with unknown pathogenicity. It is remarkable that TES technology showed a higher mutation detection rate than DNA resequencing array while also being a faster screening technique for patients with severe hypercholesterolemia.

The demand for molecular diagnosis leading to more personalized treatment of genetic diseases has increased the necessity of developing more accurate, cheaper and faster technologies to obtain genetic information. This challenge has triggered an improvement in the development of next-generation sequencing technologies (NGS). In the case of FH, Rios *et al*. first used whole-genome resequencing to perform a genetic diagnosis in an 11-month-old female with severe hypercholesterolemia and found that the patient carried two nonsense mutations in the ABCG5 gene^14^. Since then, whole exome sequencing has been used to detect mutations in FH patients, but this technology has not yet found any FH-causing mutations in genes other than LDLR, APOB or PCSK9^15^,^16^. Target exome sequencing in combination with clinical criteria now has a high success rate in the genetic diagnosis of FH patients with hyperlipidemia^8^,^17^,^18^,^19^. TES is also cheaper and more efficient than other genetic diagnostic technologies. Despite these advantages, few studies have focused on screening patients with severe hypercholesterolemia. The primary challenge in studying severe hypercholesterolaemia is the extremely low prevalence of HoFH, affecting approximately 1 in 160,000 people around the world^20^.

In the general population both HoFH and HeFH is significantly underdiagnosed. Therefore, there is a pressing clinical incentive to develop effective screening methods^4^,^20^ for the early detection of FH. In a previous study, we used whole exome sequencing and TES to identify patients with HoFH and found that these technologies are useful for providing an accurate genetic diagnosis in patients with severe hypercholesterolemia^10^,^11^. In the present work, we have analyzed 20 young patients with severe hypercholesterolemia by either array sequencing or TES. The results show that 95% are homozygous or compound heterozygous FH patients. Among them, there were 6 probands in which the genetic analysis was performed by both methods. DNA array sequencing had an overall mutation detection rate of 72.7% (8/11), while the mutation detection rate of TES was 100% (11/11). Future large cohort studies should confirm the higher mutation-detecting efficiency of TES in severe hypercholesterolemia patients.

In addition to characterizing the efficiency of TES, our study also identified 3 novel LDLR mutations and 8 mutations with previously unknown pathogenicity. Across all probands, 27 LDLR mutations were identified. *In silico* analysis predicted all of these mutations to be pathogenic. We experimentally characterized LDLR activity in the 3 novel LDLR variants (c.673delA, p.Leu575Phe and p.Leu582Phe) and one variant previously described by our group (c.1363delC). We found that all four variants are pathogenic and result in reduced LDLR expression and LDL uptake.

Through cascade screening, we then found 52 relative patients who also carried LDLR mutations. Based on their genotype, all patients were divided into three groups: HoFH, compound heterozygous and HeFH patients. In agreement with other studies, the HeFH group showed lower TC, LDL-C and non-HDL-C level than other two groups. Interestingly, in the HeFH group we found 7 patients with significantly higher levels of TC, LDL-C and non-HDL-C. One possible explanation for this finding is that LDLR activity in this subset of patients is also being modulated by epigenetic factors. A recent study found that DNA methylation plays a significant role in the clinical heterogeneity of FH patients because epigenetic perturbations of key lipoprotein metabolism genes affect plasma lipid levels in these patients^21^. We further divided HeFH patients into 3 subgroups based on the type of mutation present: missense, nonsense, and splicing-site mutations. There was only one significant difference in lipids levels among the three groups. LDL-C levels in the nonsense mutations group were significantly lower than those in the missense mutations group. There are several possible explanations for this finding: First, the sample size of this study is not big enough and needs more power to represent the entire cohort. Second, these patients may carry a disruptive-missense mutation that could influence clinical results. Previous studies have identified a class of LDLR disruptive-missense mutations that significantly elevate LDL-C levels^22^. Third, environmental factors, such as lifestyles or diet could also have had an influence on these results.

Despite the constant improvement of diagnostic tools, no causative major gene mutation has been identified in about half of all FH patients^23^. It is possible that FH has a polygenic origin for many of these patients. In 2013, Talmud *et al*. first used 12 LDL-C relative SNPs to set up a gene score which can distinguish patients with polygenic and monogenic FH from a case-control study^23^. Recently, Futema *et al*. used ROC curves to determine the optimum number of LDL-C SNPs and refined the genetic risk score using only 6 LDL-C SNPs to diagnose polygenic FH in samples^24^. In this study, we found one proband with a homozygous clinical phenotype without any mutation in LDLR, apoB100 or PCSK9. Accordingly, this patient may be a carrier of polygenic hypercholesterolemia.

In conclusion, we used TES to perform genetic diagnosis of young patients with severe hypercholesterolemia and found 27 LDLR mutations. Among them, 3 novel LDLR mutations were found and functionally characterized as pathogenic. In addition, TES technology showed the highest mutation detection rates. The results indicate that TES is a very efficient tool for genetic diagnosis in young patients with severe hypercholesterolemia.

## Materials and Methods

### Study subjects

A total of 20 unrelated patients with total cholesterol > 10 mmol/L were included in the study. Subjects attended the atherosclerosis clinic at the Anzhen hospital, Beijing, China, during the period 2003–2015. DNA samples from 12 patients were processed using DNA resequencing array^7^, samples from 14 patients were processed by targeted exome sequencing^10^, and samples from 6 patients were processed by both methods. Some probands were reported in previous studies^11^,^13^,^25^,^26^. This study was approved by the Research Ethics Committee of Beijing Anzhen Hospital in China. Written informed consent was obtained from all participants. The study was performed in accordance with approved guidelines.

### Data collection

Basic clinical data (included age, gender, family history and treatment) were recorded when patients were first admitted to the hospital. Peripheral venous blood samples were withdrawn after a 12 h fasting period. TC, LDL-c, triglycerides (TG) and high-density lipoprotein cholesterol (HDL-C) were measured using routine commercial kits (Beckman Coulter, Brea, USA) and an automated biochemistry analyzer (Beckman AU 4500, Brea, USA)^27^. Blood samples after lipid lowering treatment were also recorded for future evaluation.

### DNA extraction and sequencing

DNA was extracted by using the phenol-chloroform centrifugation method. The exon-by-exon sequencing method was used, and the Touchdown Polymerase chain reaction (PCR) method developed in our laboratory was used to process the PCR^25^. The presence of mutations in the LDLR gene, PCSK9 gene and the R3500Q mutation in the APOB gene was determined. All of the 18 exons and exon-intron boundaries of the LDLR (GeneBank accession number: NM_000527.4), the 12 exon and exon-intron boundaries of the PCSK9 gene (GeneBank accession number: NM_174936), and exon 26 and exon 29 of APOB were analyzed^27^.

### Target exome sequencing

A total of 167 lipid related genes which included genes associated blood lipids and bile acid metabolism were chosen by a previously reported gene capture strategy using a GenCap Custom Enrichment Kit (MyGenostics, Beijing, China)^10^,^28^. The targeted region included all 3386 exons of all 167 lipid-related genes and was enriched with custom-made oligonucleotide probes. The assay was performed according to the manufacturer’s protocol (MyGenostic, China). Briefly, 1 μg DNA library was mixed with a GenCap hypercholesterolemia probe (MyGenostics, China) in BL buffer, and heated at 95 °C for 7 min and 65 °C for 2 min. Then, 23 μL of the 65 °C pre-warmed HY buffer were added and held at 65 °C for 22 h to allow for hybridization. 50 μL MyOne beads (Life Technology, Carlsbad, CA) were equilibrated as indicated by the manufacturer and the eluted DNA was amplified as follows: 98 °C for 30 s; 98 °C for 25 s, 65 °C for 30 s, 72 °C for 30 s (15 cycles); 72 °C for 5 min. Then, the PCR product was purified using SPRI beads (Beckman Coulter) following the manufacturer’s protocol. Enrichment libraries were sequenced on an Illumina HiSeq 2000 sequencer (Illumina, San Diego, CA) for 100-bp paired reads.

### DNA resequencing array

The FH resequencing array was performed as previously reported by Chiou *et al*.^7^. The array was designed based on photolithography and solid-phase DNA synthesis by Vita Genomics, Inc. and manufactured by Affymetrix (Santa Clara, CA)^7^. Each microarray contained 12.6 kb in duplication of coding exon and flanking intron sequences (both sense and antisense) of the 3 most relevant FH causing genes, LDLR, APOB, and PCSK9^7^. The FH arrays were scanned using the Affymetrix GeneChip scanner 3000 7G creating CEL files for subsequent analysis^7^.

### Prediction of mutation effects

Novelty of the mutations found in this study was confirmed by searching in the PubMed and LOVD databases (www.ucl.ac.uk/ldlr/LOVDv.1.1.0/ and https://grenada.lumc.nl/LOVD2/UCLHeart/home.php?select_db = LDLR). Pathogenicity prediction of the novel mutations found in this study was assessed *in silico* using the following publicly-available softwares: PolyPhen2^29^, SIFT^30^, Mutation Taster^31^, Align GVGD (http://agvgd.iarc.fr)^32^ and Combined Annotation Dependent Depletion (CADD) (http://cadd.gs.washington.edu/)^33^. The reference sequence used for LDLR was NM_000527.3 (NCBI RefSeq).

### Cell culture and transfection for Western blot analysis of LDLR variants

A plasmid containing WT-LDLR tagged at the N-terminal with enhanced green fluorescent protein (EGFP) was constructed as previously described^27^. The mutations under study (c.673delA, c.1363delC, p.Leu575Phe and p.Leu582Phe) were introduced using a gene site-directed mutagenesis kit (Biomed, China) according to the manufacturer’s instructions. *LDLR*-deficient CHO cell line *ldl*A7 (CHO-*ldl*A7) (kindly provided by Dr. Monty Krieger, Massachusetts Institute of Technology, Cambridge, MA) was cultured in Ham’s F-12 medium supplemented with 5% FBS, 2 mM L-glutamine, 100 units/mL penicillin, and 100 μg/mL streptomycin. CHO-*ldl*A7 cells were transfected with plasmids carrying the *LDLR* variants using Lipofectamine^®^ LTX and Plus^TM^ Reagent (Invitrogen) according to the manufacturer’s instructions. Transfected cells were maintained in culture during 48 h to achieve maximal LDLR expression. Cell lysates were prepared, protein concentration determined, and fractionated by electrophoresis on non-reducing 8.5% SDS-PAGE for immunoblotting. Membranes were immunostained with rabbit polyclonal anti-LDLR antibody (1:2,000) (Progen Biotechnik GmbH, Heidelberg, Germany) for 16 h at 4 °C and anti-GAPDH antibody (1:1000) (Nordic Biosite, Täby, Sweden) for 1 h at room temperature and counterstained with a horseradish peroxidase-conjugated anti-rabbit antibody (GE Healthcare, Little Chalfont, UK). The signals were developed using SuperSignal West Dura Extended Substrate (Pierce Biotechnology, Rockford, IL, USA).

### *In vitro* functional characterization of LDLR variants

Human embryonic kidney 293 (HEK-293) cells were transiently transfected with the corresponding plasmids using Lipofectamine^®^ 3000 (Invitrogen, USA). LDLR functional studies were then performed 48 h after transfection. Flow cytometry (BD, USA) was used to determine the amount of cell-surface LDLR expression, LDL binding and internalization activities. Phycoerythrin (PE)-conjugated mouse monoclonal anti-human LDLR (1:20, R&D) antibodies were used to assay LDLR expression, and 20 μg/ml Dil-LDL (Molecular Probes, USA) was used to determine LDL binding and internalization^27^. Flow cytometry results (expression, LDL binding and LDL uptake) have been relativized to GFP signal that is used as an internal control of transfection efficiency.

### Statistical analysis

All measurements were performed at least three times in LDLR functional assay studies. Results are presented as mean ± SD. A *P*-value < 0.05 was considered statistically significant. For normally distributed continuous characteristics, the data are presented as the mean ± SD. A Student’s *t* test was used to analyze the mean values of quantitative variables. A *χ*^2^ test was used to analyze frequencies of qualitative variables. SPSS software (version 18.0 for Windows; SPSS, IBM) was used for statistical analysis.

## Additional Information

**How to cite this article**: Jiang, L. *et al*. The use of targeted exome sequencing in genetic diagnosis of young patients with severe hypercholesterolemia. *Sci. Rep*. **6**, 36823; doi: 10.1038/srep36823 (2016).

**Publisher’s note:** Springer Nature remains neutral with regard to jurisdictional claims in published maps and institutional affiliations.

## Supplementary Material

Supplementary Information

## Figures and Tables

**Table 1:** The clinical characteristics of the probands.

	Index	All Probands (n = 20)
General characteristics	Age (Years)	14.8 ± 8.8
	Male (n,%)	10 (50%)
	Origin (n,%)	South (12, 60%)
	Xanthoma (n,%)	20 (100%)
	Corneal arcus (n,%)	17 (85%)
	Chief complaint (n,%)	Xanthoma (16, 80%)
	Smoking (n,%)	3 (15%)
History	CAD (n,%)	3 (15%)
	DM (n,%)	0 (0%)
Ultrasound	MR (n,%)	10 (50%)
	AR (n,%)	11 (55%)
	AC (n,%)	15 (75%)
	IMT (cm)	0.18 ± 0.05
	CVFR	2.19 ± 0.49
Lipids before treatment	TC (mmol/L)	16.1 ± 2.9
	LDL-C (mmol/L)	13.2 ± 2.4
	TG (mmol/L)	1.5 ± 0.6
	HDL (mmol/L)	1.9 ± 0.9
Initial treatment	Statin	18 (90%)
	Statin + Ezetimibe	4 (20%)
	Xuezhikang	1 (5%)

**Table 2:** LDLR mutations identified in the study.

Number	Detected by Array	Detected by exon sequencing	Exon	cDNA	Protein
1	Yes	Yes	10	c. 1439 C > T	p. Ala480Val
	No	Yes	12	c. 1729 T > G	p. Trp577Gly
2	Yes	Yes	13	c. 1864 G > T	p. Asp622Tyr
3	Yes	ND	13	c. 1879 G > A	p. Ala627Thr
4	Yes	Yes	4	c. 611 G > A	p. Cys204Tyr
	No	Yes	4	c. 673delA	p. Lys225Asnfs*40
5	Yes	ND	4	c. 691 T > C	p. Cys231Arg
6	Yes	ND	14	c. 2054 C > T	p. Pro685Leu
7	Yes	Yes	10	c. 1448 G > A	p. Trp483X
	No	Yes	10	c. 1363delC	p. Gln455Serfs*52
8	Yes	Yes	4	c. 517 T > C	p. Cys173Arg
	Yes	Yes	12	c. 1757 C > A	p. Ser586X
9	Yes	Yes	4	c. 428 G > A	p. Cys143Tyr
	Yes	Yes	12	c. 1744 C > T	p. Leu582Phe
10	Yes	ND	10	c. 1448 G > A	p. Trp483X
	Yes	ND	13	c. 1879 G > A	p. Ala627Thr
11	No	ND	Intron 8	c. 1187-10 G > A	
12	Yes	ND	Intron 7	c. 1060 + 10 G > C	
13	ND	Yes	8	c. 1129 T > G	p. Cys377Gly
	ND	Yes	9	c. 1268 T > C	p. Ile423Thr
14	ND	Yes	Intron 5	c. 818-2 A > G	
	ND	Yes	10	c. 1448 G > A	p. Trp483X
15	ND	Yes	10	c. 1448 G > A	p. Trp483X
	ND	Yes	17	c. 2475 C > G	p. Asn825Lys
16	ND	Yes	10	c. 1448 G > A	p. Trp483X
	ND	Yes	9	c. 1216 C > A	p. Arg406Arg
17	ND	Yes	4	c. 514 G > C	p. Asp172His
	ND	Yes	12	c. 1723 C > T	p. Leu575Phe
18	ND	Yes	9	c. 1246 C > T	p. Arg416Trp
	ND	Yes	12	c. 1747 C > T	p. His583Tyr
19	ND	Yes	10	c. 1448 G > A	p. Trp483X
	ND	Yes	14	c. 2132 G > A	p. Cys711Tyr
20	ND	Yes	4	c. 665 G > T	p. Cys222Phe
	ND	Yes	14	c. 2054 C > T	p. Pro685Leu

**Table 3:** Relationship between genotype and phenotype.

	HoFH	Compound hetergyous FH	HeFH	HeFH with high TC	Mutation (−)
N	4	14	52	7	1
Age (Year)	17.7 ± 12.6	13.5 ± 6.8	38.9 ± 14.7*	38.6 ± 6.9	6
Male (%)	75%	50%	44.2%	28.6%	0%
TC	17.2 ± 2.9	16.2 ± 2.8	7.7 ± 1.6*	10.9 ± 0.9&	13.6
LDL-C	15.0 ± 3.6	13.0 ± 1.7	5.5 ± 1.7*	8.6 ± 1.5&	11.6
TG	1.8 ± 0.3	1.4 ± 0.7	1.3 ± 0.7	1.6 ± 0.2	1.6
HDL-C	1.6 ± 1.0	1.9 ± 0.9	1.4 ± 0.5	1.7 ± 0.5	1.2
Non-HDL-C	15.6 ± 3.2	14.3 ± 2.6	6.3 ± 1.5*	9.3 ± 1.0&	12.3

^*^The significant difference between heterozygous FH patients and either homozygous FH or compound heterozygous FH patients.

^&^The significant difference between the HeFH with high TC group and other HeFH patients group.
