# Supplementary material for: The use of targeted exome sequencing in genetic diagnosis of young patients with severe hypercholesterolemia
Source: Sci Rep. 2016 Nov 10;6:36823. doi: 10.1038/srep36823 (PMC5103295; doi:10.1038/srep36823)
Supplement: Supplementary Information [file srep36823-s1.doc]

**The use of targeted exome sequencing in genetic diagnosis of young patients with severe hypercholesterolemia**

Long Jiang, MD, PhD^1,2#^, Wen-Feng Wu, MD^1#^, Li-Yuan Sun, MD^4^, Pan-Pan Chen, MS^1,5^, Wei Wang, MS^1,6^, Xiao-Dong Pan, MD^1^, Wei Cui, MD^1^, Shi-Wei Yang, MD^1^, Yu-Jie Zhou, PhD^1^, Cesar Martin, PhD^7^, and Lu-Ya Wang, PhD^1^*

**Supplements**

**Table S1 All the mutations in this study.**

**Table S2 The *in silio* analysis of both novel mutations and unknown pathogenicity mutations**

**Table S3 The clinical characteristic of HeFH patients with different genotype.**

**Table S1 All the mutations in this study.**

| Exon | cDNA | Protein | LDLR Activity | Populations |
| --- | --- | --- | --- | --- |
| Exon 4 | c. 428 G>A | p. Cys143Tyr | About 93% LDLR expression, 59% binding and 73% internalizing activities left when transfected into 293T cells | China[^1^](#_ENREF_1); Germany |
| Exon 4 | c. 514 G>C | p. Asp172His | NA | South Africa[^4^](#_ENREF_4) |
| Exon 4 | c. 517 T>C | p. Cys173Arg | 5-15% LDLR activity when heterozygous with FH Greece | Greek[^5-7^](#_ENREF_5) |
| Exon 4 | c. 611 G>A | p. Cys204Tyr | NA | Italy[^8^](#_ENREF_8) |
| Exon 4 | c. 665 G>T | p. Cys222Phe | NA | Netherlands[^9^](#_ENREF_9) |
| Exon 4 | c. 673delA | p. Lys225Asnfs*40 | NA | NA |
| Exon 4 | c. 691 T>C | p. Cys231Arg | About 89% LDLR expression, 56% binding and 62% internalizing activities left when transfected into 293T cells | China[^1^](#_ENREF_1) |
| Intron 5 | c. 818-2 A>G |  | NA | Portugal[^10^](#_ENREF_10) |
| Intron 7 | c. 1060+10 G>C |  | Estimated splicing modification negative | France[^11^](#_ENREF_11); Greece[^3^](#_ENREF_3); UK[^12^](#_ENREF_12); Danish[^13^](#_ENREF_13); Denmark[^14^](#_ENREF_14); Malaysia[^15^](#_ENREF_15); Portugal[^16^](#_ENREF_16) |
| Exon 8 | c. 1129 T>G | p. Cys377Gly | About 57% binding and 62% internalizing activities left when transfected into 293T cells | China[^17^](#_ENREF_17) |
| Intron 8 | c. 1187-10 G>A |  | Resulted in a transcript which had the eight last nucleotides of intron 8 included in cDNA and left 22% normal transcript in EBV-transformed lymphocytes from heterozygous patients | China[^18^](#_ENREF_18); Canada[^19^](#_ENREF_19); France[^11^](#_ENREF_11); Philippines[^20^](#_ENREF_20); Norway[^21^](#_ENREF_21) |
| Exon 9 | c. 1216 C>A | p. Arg406Arg | It causes a deletion of 31 bp from the mRNA, and was predicted to introduce premature termination four codons after R406 | China; Portugal[^24^](#_ENREF_24); UK[^25^](#_ENREF_25) |
| Exon 9 | c. 1246 C>T | p. Arg416Trp | NA | Austria[^26^](#_ENREF_26); Czech Republic[^27^](#_ENREF_27); China[^28^](#_ENREF_28); UK[^29^](#_ENREF_29); Spain[^30^](#_ENREF_30); German; Norway[^26^](#_ENREF_26); Netherland[^9^](#_ENREF_9); Poland[^31^](#_ENREF_31) |
| Exon 9 | c. 1268 T>C | p. Ile423Thr | 54% LDLR activity in transfected COS cells | Sweden[^32^](#_ENREF_32); Malaysia[^33^](#_ENREF_33); China[^17^](#_ENREF_17); |
| Exon 10 | c. 1363delC | p. Gln455Serfs*52 | NA | China[^23^](#_ENREF_23) |
| Exon 10 | c. 1439 C>T | p. Ala480Val | 39% LDLR expression, 63% binding and 76% internalization activity in peripheral blood lymphocytes from HoFH patients | China[^34^](#_ENREF_34) |
| Exon 10 | c. 1448 G>A | p. Trp483X | 17% LDLR binding and 39% internalization activity in transfected 293T cells | China[^35^](#_ENREF_35); Austria[^26^](#_ENREF_26) |
| Exon 12 | c. 1723 G>T | p. Leu575Phe | NA | NA |
| Exon 12 | c. 1729 T>G | p. Trp577Gly | <20% LDLR activity in transfected CHO cells | Austria[^36^](#_ENREF_36); Spain[^37^](#_ENREF_37) |
| Exon 12 | c. 1744 C>T | p. Leu582Phe | NA | NA |
| Exon 12 | c. 1747 C>T | p. His583Tyr | Precursor accumulates; approximately 50% mature protein | Austria[^38^](#_ENREF_38); China[^35^](#_ENREF_35); Philippines[^39^](#_ENREF_39) |
| Exon 12 | c. 1757 C>A | p. Ser586X | 16% LDLR binding and 19% internalization activity in transfected 293T cells | China[^40^](#_ENREF_40) |
| Exon 13 | c. 1864 G>T | p. Asp622Tyr | 13.6% LDLR expression and 21.1% binding activity in peripheral blood lymphocytes from patients | China[^41^](#_ENREF_41) |
| Exon 13 | c. 1879 G>A | p. Ala627Thr | Bind LDL slow processing recycling defective | China[^41^](#_ENREF_41) |
| Exon 14 | c. 2054 C>T | p. Pro685Leu | 15-30% LDLR activity in homozygote | UK[^42^](#_ENREF_42); Japan[^43^](#_ENREF_43); Netherlands[^44^](#_ENREF_44); China[^41^](#_ENREF_41) |
| Exon 14 | c. 2132 G>A | p. Cys711Tyr | NA | China[^23^](#_ENREF_23); Malaysia[^45^](#_ENREF_45) |
| Exon 17 | c. 2475 C>G | p. Asn825Lys | NA | China[^23^](#_ENREF_23); Denmark[^46^](#_ENREF_46); Netherlands[^9^](#_ENREF_9); Canada[^19^](#_ENREF_19); Spain[^47^](#_ENREF_47) |

*

**Table S2 The *in silio* analysis of both novel mutations and unknown pathogenicity mutations**

| Exon | cDNA | Protein | Align GVGD^*^ | Pathogenicity prediction | | | CADD  (PHRED Value)^&^ |
| --- | --- | --- | --- | --- | --- | --- | --- |
|  |  |  |  | Polyphen-2 | SIFT | Mutation taster |  |
| Exon 4 | c. 514 G>C | p. Asp172His (D172H) | Class C65 | Probably damaging (1) | Not tolerated | Disease causing | 10.83 |
| Exon 4 | c. 611 G>A | p. Cys204Tyr (C204Y) | Class C65 | Probably damaging (1) | Not tolerated | Disease causing | 10.83 |
| Exon 4 | c. 665 G>T | p. Cys222Phe (C222F) | Class C65 | Probably damaging(1) | Not tolerated | Disease causing | 7.147 |
| Exon 4 | c. 673delA | p. Lys225Asnfs*40 | Class C65 | - | - | Disease causing | - |
| Intron 5 | c. 818-2 A>G |  |  |  |  |  | - |
| Exon 9 | c. 1246 C>T | p. Arg416Trp (R416W) | Class C65 | Probably damaging (0.998) | Not tolerated | Disease causing | 11.81 |
| Exon 10 | c. 1363delC | p. Gln455Serfs*52 | Class C65 | - | - | Disease causing | - |
| Exon 12 | c. 1723 C>T | p. Leu575Phe (L575F) | Class C15 | Probably damaging (1) | Not tolerated | Disease causing | 12.34 |
| Exon 12 | c. 1744 C>T | p. Leu582Phe (L582F) | Class C15 | Probably damaging (0.991) | Not tolerated | Disease causing | 11.69 |
| Exon 14 | c. 2132 G>A | p. Cys711Tyr (C711Y) | Class C65 | Probably damaging (1) | Not tolerated | Disease causing | 16.05 |
| Exon 17 | c. 2475 C>G | p. Asn825Lys (N825K) | Class C65 | Probably damaging (1) | Not tolerated | Disease causing | 21.3 |

* Score values are from C0 (not pathogenic) to C65 (pathogenic).

^&^ PHRED-like (_10*log10(rank/total)) scaled C-score ranking a variant relative to all possible substitutions of the human genome. No cut-off is automatically established by the program but they recommend 15 or, at least, higher than 10.

**Table S3 The clinical characteristic of HeFH patients with different genotype.**

|  | Patients with missense mutations | Patients with nonsense mutations | Patients with splicing-site mutations |
| --- | --- | --- | --- |
| **N** | 35 | 12 | 5 |
| **Age (Year)** | 42.1±13.4 | 31.4±17.0* | 35±12.6 |
| **Male (%)** | 42.9% | 58.3% | 20% |
| **TC** | 7.9±1.8 | 7.3±1.0 | 7.6±1.6 |
| **LDL-C** | 5.8±1.9 | 5.0±0.8* | 4.8±1.2 |
| **TG** | 1.4±0.7 | 1.2±0.7 | 1.2±0.5 |
| **HDL-C** | 1.4±0.5 | 1.5±0.5 | 1.4±0.2 |
| **Non-HDL-C** | 6.5±1.7 | 5.8±0.8 | 6.1±1.7 |

* The significant different between patients with nonsense mutations group and missense mutations group.

**References**

1. Cao, S.*, et al.* Analysis of low-density lipoprotein receptor gene mutations in a Chinese patient with clinically homozygous familial hypercholesterolemia. *Chin Med J (Engl)* **116**, 1535-1538 (2003).

2. Genschel, J.*, et al.* Two novel LDL receptor mutations in familial hypercholesterolemia: C122Y and E296X. *Hum Mutat* **17**, 354 (2001).

3. Dedoussis, G.V.*, et al.* Molecular characterization of familial hypercholesterolemia in German and Greek patients. *Hum Mutat* **23**, 285-286 (2004).

4. Thiart, R.*, et al.* Predominance of a 6 bp deletion in exon 2 of the LDL receptor gene in Africans with familial hypercholesterolaemia. *J Med Genet* **37**, 514-519 (2000).

5. Miltiadous, G.*, et al.* Characterization and geographic distribution of the low density lipoprotein receptor (LDLR) gene mutations in northwestern Greece. *Hum Mutat* **17**, 432-433 (2001).

6. Mollaki, V., Progias, P. & Drogari, E. Familial Hypercholesterolemia in Greek children and their families: genotype-to-phenotype correlations and a reconsideration of LDLR mutation spectrum. *Atherosclerosis* **237**, 798-804 (2014).

7. Diakou, M.*, et al.* Spectrum of LDLR gene mutations, including a novel mutation causing familial hypercholesterolaemia, in North-western Greece. *Eur J Intern Med* **22**, e55-59 (2011).

8. Cefalu, A.B.*, et al.* Six novel mutations of the LDL receptor gene in FH kindred of Sicilian and Paraguayan descent. *Int J Mol Med* **17**, 539-546 (2006).

9. Fouchier, S.W., Defesche, J.C., Umans-Eckenhausen, M.W. & Kastelein, J.P. The molecular basis of familial hypercholesterolemia in The Netherlands. *Hum Genet* **109**, 602-615 (2001).

10. Bourbon, M.*, et al.* Genetic diagnosis of familial hypercholesterolaemia: the importance of functional analysis of potential splice-site mutations. *J Med Genet* **46**, 352-357 (2009).

11. Amsellem, S.*, et al.* Intronic mutations outside of Alu-repeat-rich domains of the LDL receptor gene are a cause of familial hypercholesterolemia. *Hum Genet* **111**, 501-510 (2002).

12. Sozen, M.M.*, et al.* The molecular basis of familial hypercholesterolaemia in Turkish patients. *Atherosclerosis* **180**, 63-71 (2005).

13. Brusgaard, K., Jordan, P., Hansen, H., Hansen, A.B. & Horder, M. Molecular genetic analysis of 1053 Danish individuals with clinical signs of familial hypercholesterolemia. *Clin Genet* **69**, 277-283 (2006).

14. Larsen, M.K., Nissen, P.H., Kristensen, I.B., Jensen, H.K. & Banner, J. Sudden cardiac death in young adults: environmental risk factors and genetic aspects of premature atherosclerosis. *J Forensic Sci* **57**, 658-662 (2012).

15. Al-Khateeb, A.*, et al.* Analysis of sequence variations in low-density lipoprotein receptor gene among Malaysian patients with familial hypercholesterolemia. *BMC Med Genet* **12**, 40 (2011).

16. Cymbron, T.*, et al.* Familial hypercholesterolemia: Molecular characterization of possible cases from the Azores Islands (Portugal). *Meta Gene* **2**, 638-645 (2014).

17. Wu, W.F., Sun, L.Y., Pan, X.D., Yang, S.W. & Wang, L.Y. Use of targeted exome sequencing in genetic diagnosis of chinese familial hypercholesterolemia. *PLoS ONE* **9**(2014).

18. Sun, L.Y.*, et al.* Identification of the gene defect responsible for severe hypercholesterolaemia using whole-exome sequencing. *Sci Rep* **5**, 11380 (2015).

19. Wang, J., Huff, E., Janecka, L. & Hegele, R.A. Low density lipoprotein receptor (LDLR) gene mutations in Canadian subjects with familial hypercholesterolemia, but not of French descent. *Hum Mutat* **18**, 359 (2001).

20. Punzalan, F.E.*, et al.* Low density lipoprotein--receptor (LDL-R) gene mutations among Filipinos with familial hypercholesterolemia. *J Atheroscler Thromb* **12**, 276-283 (2005).

21. Holla, O.L.*, et al.* Effects of intronic mutations in the LDLR gene on pre-mRNA splicing: Comparison of wet-lab and bioinformatics analyses. *Mol Genet Metab* **96**, 245-252 (2009).

22. Fan, L.L.*, et al.* Novel mutations of low-density lipoprotein receptor gene in China patients with familial hypercholesterolemia. *Appl Biochem Biotechnol* **176**, 101-109 (2015).

23. Jiang, L.*, et al.* Characterization of the unique Chinese W483X mutation in the low-density lipoprotein–receptor gene in young patients with homozygous familial hypercholesterolemia. *J Clin Lipidol.* (2015).

24. Bourbon, M.*, et al.* Familial hypercholesterolaemia in Portugal. *Atherosclerosis* **196**, 633-642 (2008).

25. Bourbon, M., Sun, X.M. & Soutar, A.K. A rare polymorphism in the low density lipoprotein (LDL) gene that affects mRNA splicing. *Atherosclerosis* **195**, e17-20 (2007).

26. Schmidt, H. & Kostner, G.M. Familial hypercholesterolemia in Austria reflects the multi-ethnic origin of our country. *Atherosclerosis* **148**, 431-432 (2000).

27. Duskova, L.*, et al.* An APEX-based genotyping microarray for the screening of 168 mutations associated with familial hypercholesterolemia. *Atherosclerosis* **216**, 139-145 (2011).

28. Han, Y.*, et al.* Clinical features of bilateral temporal bone xanthoma with LDLR gene mutation. *Int J Pediatr Otorhinolaryngol* **79**, 1148-1151 (2015).

29. Day, I.N.*, et al.* Spectrum of LDL receptor gene mutations in heterozygous familial hypercholesterolemia. *Hum Mutat* **10**, 116-127 (1997).

30. Garcia-Garcia, A.B.*, et al.* Molecular genetics of familial hypercholesterolemia in Spain: Ten novel LDLR mutations and population analysis. *Hum Mutat* **18**, 458-459 (2001).

31. Ahmed, W.*, et al.* The genetic spectrum of familial hypercholesterolemia in Pakistan. *Clin Chim Acta* **421**, 219-225 (2013).

32. Ekstrom, U., Abrahamson, M., Sveger, T., Lombardi, P. & Nilsson-Ehle, P. An efficient screening procedure detecting six novel mutations in the LDL receptor gene in Swedish children with hypercholesterolemia. *Hum Genet* **96**, 147-150 (1995).

33. Khoo, K.L.*, et al.* Low-density lipoprotein receptor gene mutations in a Southeast Asian population with familial hypercholesterolemia. *Clin Genet* **58**, 98-105 (2000).

34. Lin, J.*, et al.* Functional analysis of low-density lipoprotein receptor in homozygous familial hypercholesterolemia patients with novel 1439 C-->T mutation of low-density lipoprotein receptor gene. *Chin Med J (Engl)* **121**, 776-781 (2008).

35. Sun, X.M.*, et al.* Familial hypercholesterolemia in China: Identification of mutations in the LDL-receptor gene that result in a receptor-negative phenotype. *Arteriosclerosis and Thrombosis* **14**, 85-94 (1994).

36. Widhalm, K., Dirisamer, A., Lindemayr, A. & Kostner, G. Diagnosis of families with familial hypercholesterolaemia and/or Apo B-100 defect by means of DNA analysis of LDL-receptor gene mutations. *J Inherit Metab Dis* **30**, 239-247 (2007).

37. Etxebarria, A.*, et al.* Functional characterization and classification of frequent low-density lipoprotein receptor variants. *Hum Mutat* **36**, 129-141 (2015).

38. Hooper, A.J.*, et al.* Genetic analysis of familial hypercholesterolaemia in Western Australia. *Atherosclerosis* **224**, 430-434 (2012).

39. Punzalan, F.E.*, et al.* Low density lipoprotein--receptor (LDL-R) gene mutations among Filipinos with familial hypercholesterolemia. *J Atheroscler Thromb* **12**, 276-283 (2005).

40. Su, P.*, et al.* A novel mutation of the LDL receptor gene leading to familial hypercholesterolemia. *Eur. J. Lipid Sci. Technol.* **111**, 646-651 (2009).

41. Jiang, L.*, et al.* The distribution and characteristics of LDL receptor mutations in China: A systematic review. *Sci Rep* **5**, 17272 (2015).

42. Soutar, A.K., Knight, B.L. & Patel, D.D. Identification of a point mutation in growth factor repeat C of the low density lipoprotein-receptor gene in a patient with homozygous familial hypercholesterolemia that affects ligand binding and intracellular movement of receptors. *Proc Natl Acad Sci U S A* **86**, 4166-4170 (1989).

43. Maruyama, T.*, et al.* Common mutations in the low-density-lipoprotein-receptor gene causing familial hypercholesterolemia in the Japanese population. *Arterioscler Thromb Vasc Biol* **15**, 1713-1718 (1995).

44. Defesche, J.C.*, et al.* Detection of the Pro664-Leu mutation in the low-density lipoprotein receptor and its relation to lipoprotein(a) levels in patients with familial hypercholesterolemia of Dutch ancestry from The Netherlands and Canada. *Clin Genet* **42**, 273-280 (1992).

45. Chahil, J.K., Lye, S.H., Bagali, P.G. & Alex, L. A novel pathogenic variant of the LDLR gene in the Asian population and its clinical correlation with familial hypercholesterolemia. *Mol Biol Rep* **39**, 7831-7838 (2012).

46. Jensen, H.K.*, et al.* Spectrum of LDL receptor gene mutations in Denmark: implications for molecular diagnostic strategy in heterozygous familial hypercholesterolemia. *Atherosclerosis* **146**, 337-344 (1999).

47. Alonso, R.*, et al.* Genetic diagnosis of familial hypercholesterolemia using a DNA-array based platform. *Clin Biochem* **42**, 899-903 (2009).
